# Newly Constructed Network Models of Different WNT Signaling Cascades Applied to Breast Cancer Expression Data

**DOI:** 10.1371/journal.pone.0144014

**Published:** 2015-12-03

**Authors:** Michaela Bayerlová, Florian Klemm, Frank Kramer, Tobias Pukrop, Tim Beißbarth, Annalen Bleckmann

**Affiliations:** 1 Department of Medical Statistics, University Medical Center Göttingen, Göttingen, Germany; 2 Department of Hematology and Medical Oncology, University Medical Center Göttingen, Göttingen, Germany; 3 Department of Internal Medicine III, University Hospital Regensburg, Regensburg, Germany; University of Washington, UNITED STATES

## Abstract

**Introduction:**

WNT signaling is a complex process comprising multiple pathways: the canonical β-catenin-dependent pathway and several alternative non-canonical pathways that act in a β-catenin-independent manner. Representing these intricate signaling mechanisms through bioinformatic approaches is challenging. Nevertheless, a simplified but reliable bioinformatic WNT pathway model is needed, which can be further utilized to decipher specific WNT activation states within e.g. high-throughput data.

**Results:**

In order to build such a model, we collected, parsed, and curated available WNT signaling knowledge from different pathway databases. The data were assembled to construct computationally suitable models of different WNT signaling cascades in the form of directed signaling graphs. This resulted in four networks representing canonical WNT signaling, non-canonical WNT signaling, the inhibition of canonical WNT signaling and the regulation of WNT signaling pathways, respectively. Furthermore, these networks were integrated with microarray and RNA sequencing data to gain deeper insight into the underlying biology of gene expression differences between MCF-7 and MDA-MB-231 breast cancer cell lines, representing weakly and highly invasive breast carcinomas, respectively. Differential genes up-regulated in the MDA-MB-231 compared to the MCF-7 cell line were found to display enrichment in the gene set originating from the non-canonical network. Moreover, we identified and validated differentially regulated modules representing canonical and non-canonical WNT pathway components specific for the aggressive basal-like breast cancer subtype.

**Conclusions:**

In conclusion, we demonstrated that these newly constructed WNT networks reliably reflect distinct WNT signaling processes. Using transcriptomic data, we shaped these networks into comprehensive modules of the genes implicated in the aggressive basal-like breast cancer subtype and demonstrated that non-canonical WNT signaling is important in this context. The topology of these networks can be further refined in the future by integration with complementary data such as protein-protein interactions, in order to gain greater insight into signaling processes.

## Introduction

The WNT signaling pathway plays an important role not only in embryonic development processes but also in adult tissue homeostasis and repair after injury. Furthermore, its aberrant regulation is involved in a range of human pathologies such as cancer and degenerative diseases [1]. The widespread effects of WNT activity are mirrored by the pathway’s complexity–WNT signals are channeled through several distinct cascades: the canonical β-catenin-dependent pathway and several non-canonical, i.e. β-catenin-independent pathways. There is a great degree of crosstalk between these branches and interactions with a variety of external pathways [2,3]. Capturing and describing these functional relationships in an organized and comprehensive fashion in order to represent them in models suitable for bioinfomatic analysis is a challenge.

Currently, there are a number of WNT signaling pathways in public databases, such as BioCarta [4], Reactome [5], the Kyoto Encyclopedia of Genes and Genomes (KEGG) [6] and the Pathway Interaction Database (PID) [7]. How in detail the signaling pathways are modeled and the way they can be exported differs between the databases. One of the generally accepted standards is the BioPAX format, which aims to represent biological pathways at the level of basic cellular processes [8]. The WNT pathway encoded in this format can be further transformed to enable the integration of pathway data into further analyses [9]. However, even within the BioPAX definition, the scope and granularity of pathway information in the different databases varies widely. Therefore, distinct WNT signaling pathways are represented to differing extents in the databases. In most of these databases, there is only general representation of WNT signaling, either without any clear discrimination between distinct WNT sub-pathways or reflecting only canonical β-catenin-dependent signaling. Only recently were there more focused efforts, most notably by PID [7] and Reactome [5], to assemble and publish specific pathways for non-canonical WNT signaling in addition.

In brief, the canonical WNT pathway is defined by the nuclear translocation of β-catenin, in which it functions as a co-activator of transcription. In the absence of WNT signaling, β-catenin is targeted by a destruction complex composed of AXIN, APC, and GSK3β leading to its degradation by the proteasome. Binding of a WNT ligand to a frizzled (Fz) receptor and its co-receptors LRP5/6 activates intracellular dishevelled (DVL), leading to inactivation of the β-catenin destruction complex. This in turn leads to the stabilization of β-catenin, its relocation into the nucleus, and triggers the expression of target genes facilitated by TCF/LEF1 transcription factors [10,11]. This transcriptional activity governs cell proliferation and survival as well as controlling decisions on cells’ fate.

However, within the WNT signaling pathway, several other β-catenin-independent mechanisms exist that are generally defined by a lack of β-catenin-mediated transcription. Broadly, these cascades can be grouped into planar cell polarity (PCP) and WNT/Ca^2+^ signaling, representing the non-canonical WNT pathway. On the one hand this pathway can result in the activation of small GTPases such as Rho, Rac, and Cdc42, which regulate cytoskeleton rearrangement and PCP. On the other hand, it can also regulate transcription by activating the transcription factors NFAT or AP-1. Generally speaking, the non-canonical WNT pathway is linked to changes in cell motility, cytoskeletal reorganization processes, and invasiveness [3].

While the separation between these β-catenin-dependent and -independent pathways has proven valuable in understanding the signaling output, there is a high degree of interaction between these different signaling components on several levels of the cascades: The WNT protein family in humans comprises 19 members, all of which appear to be neither intrinsically canonical nor non-canonical. It is rather the multitude of possible combinations between WNT ligands and the respective Fz receptors and non-Fz (co)-receptors such as ROR1, ROR2, and RYK that channel WNT signals into one of the WNT sub-pathways [12]. Appropriately, these distinct ligand-receptor interactions lead to the transcription of different but often overlapping sets of target genes [13]. Further downstream, DVL is able to act as a key transducer of signals into each of the different WNT pathways. Finally, there is evidence of interaction at the transcriptional level, as the non-canonical WNT pathways are able to inhibit β-catenin-mediated transcription. Ultimately, these WNT signaling sub-pathways should thus not be considered as straightforward linear cascades but rather as complex overlapping signaling networks [14].

This unclear distinction of WNT signals and their consequences are illustrated by the example of breast cancer. The inappropriate activity of both canonical and non-canonical WNT pathways has been implicated in breast cancer initiation as well as progression [15]. Breast tumors are very diverse and can be categorized according to their expression patterns into five molecular subtypes: basal-like, ERBB2-overexpressing, luminal A and B, and normal-breast-like [16]. These molecular subtypes are associated with different clinical prognoses [17] and vary in their expression of WNT pathway members. The basal-like subtype in particular has been shown to exhibit enrichment of a number of WNT pathway players [18].

The particular WNT pathway involved in this enrichment has so far eluded detection. On the one hand, there is evidence pointing to the activation of β-catenin-dependent WNT signaling in basal-like breast cancer primaries [19,20]. On the other hand, non-canonical WNT signaling genes such as ROR1 were found to be overexpressed in basal-like MDA-MB-231 cells [21,22] and breast cancer brain metastases [22]. Furthermore, the expression of ROR2 was linked to shorter overall survival compared to patients with no ROR2 expression [23].

Phenotypic differences such as invasiveness are often attributed to differences in the transcriptome between the investigated populations. By comparing their expression profiles differentially expressed genes (DEGs) are identified, which can result in fairly long lists. To reduce the complexity of these lists of DEGs and unravel the underlying biological processes, gene-set enrichment or overrepresentation analysis is a widely utilized bioinformatic approach, which allows the identification of the most affected pathways [24,25]. Another popular bioinformatic approach is to explore how DEGs interact and regulate each other within the framework of molecular network analysis [26].

Prior knowledge representing the pathway is required in order to test for enrichment of differential genes in a certain pathway or to integrate expression data into a pathway network [9]. Several pathway databases systematically collect pathway data [27] and make them publicly available in the form of gene sets or also in a more complex but computer-interpretable format such as BioPAX. There is a need for a simplified but reliable model of the WNT pathways suitable for bioinformatic analysis to better understand the underlying biology of gene expression data.

Thus the aims of this study can be considered as four-fold: 1) We seek to collect available WNT signaling data across different pathway databases in the computer-readable BioPAX format to allow semi-automatized processing. 2) We aim to assemble this knowledge into directed graphs representing signaling networks of distinct WNT sub-pathways. 3) To demonstrate that these newly constructed WNT networks reliably reflect distinct WNT signaling processes, we evaluate them in an application example in the context of WNT regulation in breast cancer. Based on our previous work [22] we generated the hypothesis that especially in basal-like breast cancer the non-canonical WNT pathway is critical for cancer progression and metastasis. Juxtaposing luminal A MCF-7 and basal-like MDA-MB-231 cells we aim to evaluate differences in the regulation of WNT pathways based on the respective expression profiles. Specific differentially regulated WNT modules are then unraveled by integrating our constructed WNT networks with the gene expression data. 4) Finally, we assess the validity of the identified modules and their important elements in an independently generated dataset of RNA sequencing (RNA-Seq) profiles of the MCF-7 and MDA-MB-231 cell lines.

## Materials and Methods

### Construction of the networks

In order to assemble public pathway knowledge into network models of WNT signaling pathways three steps were taken: 1) Pathway data were gathered across several databases and parsed. 2) Gene IDs were mapped on pathway graphs. 3) The graphs were sorted and merged into four networks.

#### Pathway databases and parsing

**Fig 1:**
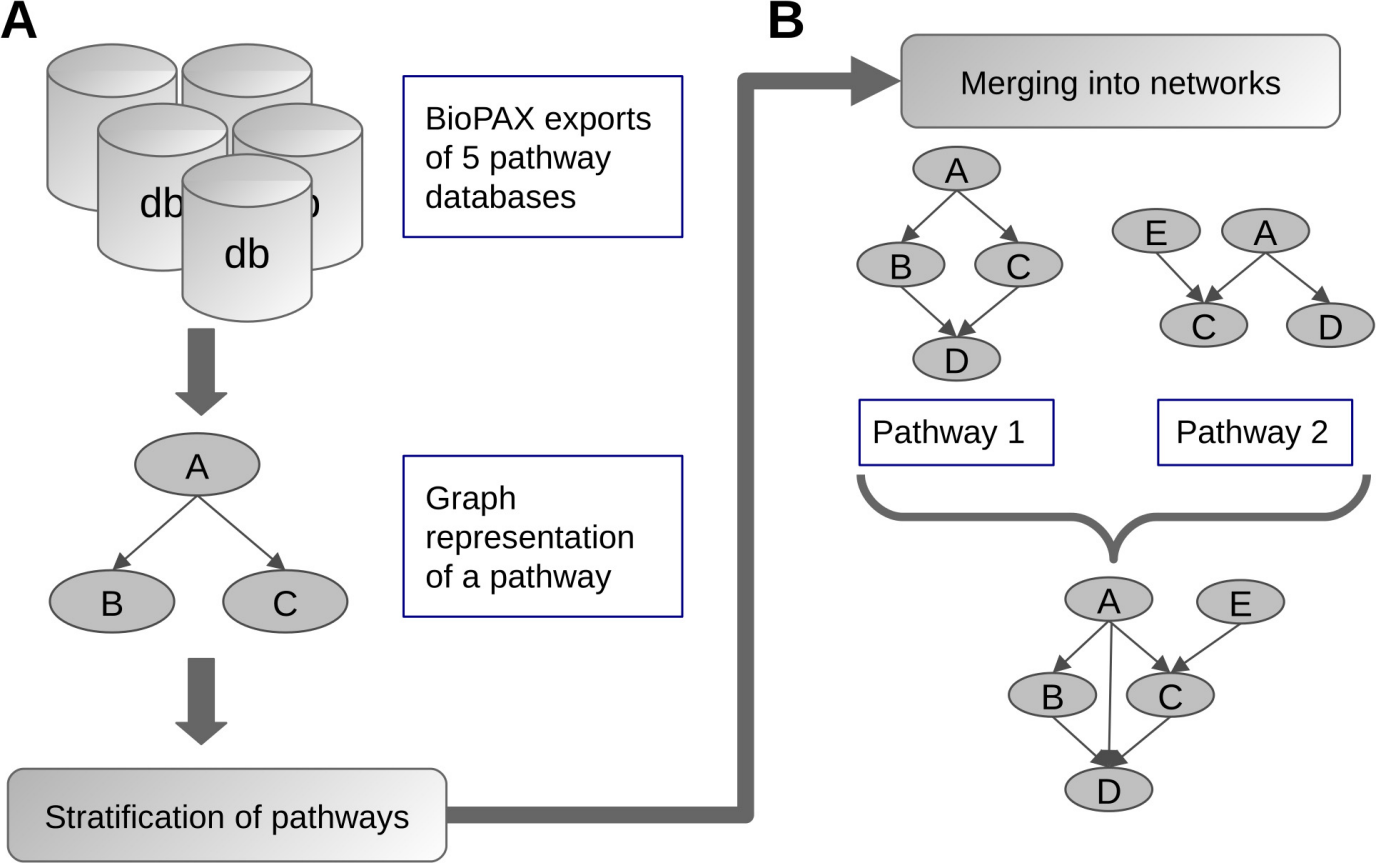
Workflow of parsing and merging pathways. BioPAX encoded pathway data on WNT signaling was collected across 5 databases, parsed into R, and transformed into signaling graphs (A). The pathways were stratified into conceptual groups and within each group the graphs were sequentially merged into a signaling network. When merging two pathways, the resulting graph contains all nodes and edges from both original graphs (B).

Five pathway databases were used to collect WNT signaling data: BioCarta [4], Reactome [5], Kyoto Encyclopedia of Genes and Genomes (KEGG) [6], Pathway Interaction Database (PID) [7] and Pathway Commons [28]. The database exports of BioPAX level 3 files were downloaded in March 2014 and parsed into R using the *rBiopaxParser* R-package [29]. Pathways of interest were selected according to the presence of key words such as 'wnt', 'catenin', and 'pcp' either within pathway names or their members. The pathways were then transformed into interaction graphs in which the nodes represent genes [30] (Fig 1A). These interaction graphs focus specifically on controlling interactions of physical entities, i.e. the directed edges represent activation or inhibition processes between the genes. Encoded knowledge on binding mechanisms and conversions of physical entities are not represented within these graphs.

#### Gene IDs mapping

The pathway databases use different identifications to annotate pathway molecules. In most cases Entrez Gene or UniProt IDs were available as reference annotations for a node and these were converted into HUGO Gene Nomenclature Committee (HGNC) gene symbols. However, whenever no official ID was available, we chose the instance name property defined within the BioPAX model to represent a node label. Pathway entities that could not be mapped into gene expression data, such as small molecules, DNA, RNA, or nested pathways, were filtered out, while connectivity of the graphs was preserved by introducing new edges connecting the genes that were thus indirectly connected. Further, if multiple gene symbols were mapped onto a single node, such a node was split into multiple nodes and each one was assigned with a single gene symbol. Vice versa, if several nodes were annotated with the same gene symbol, these nodes were merged into one.

#### Pathway stratification and merging into networks

After the parsing and ID mapping steps, we curated pathways based both on our own knowledge and on the literature into four groups representing: canonical WNT signaling, non-canonical WNT signaling, inhibition of canonical WNT signaling, and regulation of WNT signaling. Pathways considered to be too general or unspecific to be classified were discarded. The *Wnt signaling pathway* we exported from the KEGG database contained canonical as well as non-canonical sub-pathways and therefore the corresponding connected components of the pathway graph were sorted into canonical WNT signaling and non-canonical WNT signaling groups, respectively (Fig 1B).

In order to assemble these pathways into signaling networks, each pathway graph was transformed into the simple interaction format, excluding nodes without any interactions. Next, the interaction tables were concatenated according to the assigned groups and duplicated interactions were removed. Finally, the four interaction tables were transformed back into graph objects.

### Community detection and centralities

To determine community structure in a graph we sought dense subgraphs using the fast greedy modularity optimization algorithm implemented in the *fastgreedy*.*community* R-function [31]. In order to identify network key nodes, two network centrality measures were used: betweenness centrality, defined as the number of shortest paths going through a given node, implemented in the *betweenness* R-function and degree centrality, defined for a given node as the number of its edges, implemented in the *degree* R-function. The R-functions are available in the *igraph* R-package [32].

### Microarray data and differential analysis

Public microarray data was retrieved from the NCBI Gene Expression Omnibus (GEO) data repository [33]. Three MCF-7 and two MDA-MB-231 samples of breast cancer cell lines were selected from the GSE5823 dataset [34]. Expression levels were computed using robust multi‐array average measure [35] and quantile normalized. Probes without available gene annotation were filtered out. Differential expression analysis was performed by fitting linear models using the empirical Bayes method as implemented in the limma R-package [36]. The p-values were adjusted for multiple testing using the Bonferroni correction [37]. In order to summarize probes onto the gene level we kept the probe with the lowest p-value to represent a gene.

### Treatment of cell lines

The human breast cancer cell line MCF-7 was purchased from the German Collection of Microorganisms and Cell Cultures (DSMZ, Braunschweig, Germany) and the MDA-MB-231 breast cancer cell war purchased from the American Type Culture Collection (ATCC, Rockville, USA). Both cell lines were maintained in RPMI-1640 medium (PAA Laboratories Inc., Cölbe, Germany) supplemented with 10% heat-inactivated fetal calf serum (FCS, Invitrogen, Karlsruhe, Germany). For gene expression studies, cells were seeded on extracellular matrix (R&D Systems, Wiesbaden, Germany)-coated tissue culture wells. Total RNA was isolated using TRIZOL reagent according to the manufacturer’s instructions (Invitrogen, Carlsbad, CA). RNA integrity for each sample was confirmed with the Agilent 2100 Bioanalyzer (Agilent Technologies, Palo Alto, CA).

### RNA-Seq data generation

Library preparation for RNA-Seq was performed using the TruSeq RNA Sample Preparation Kit (Illumina, Cat. N°RS-122-2002) starting from 1000 ng of total RNA according to Illumina's instructions. Accurate quantitation of cDNA libraries was performed by using the QuantiFluor™ dsDNA System (Promega). The size range of final cDNA libraries was determined applying the DNA 1000 chip on the Bioanalyzer 2100 from Agilent (280 bp). The purified DNA was captured on an Illumina flow cell for cluster generation. Libraries were sequenced on the HiSeq2000 following the manufacturer's protocols. Illumina Casava 1.8.2 software used for base-calling.

### RNA-Seq data analysis

Quality checking of RNA-Seq data was done via FastQC (v. 0.10.0, Babraham Bioinformatics). Alignment to the human transcriptome based on the ENSEMBL genome (version GRCh38 release 78) was performed using the STAR tool version 2.4.0 [38]. Expected counts for gene-level abundances were estimated by the RSEM algorithm version 1.2.19 [39]. Lowly expressed genes were filtered out by keeping the genes with at least one read count-per-million in at least two samples. Read counts were normalized to the effective library size using the weighted trimmed mean of M-values method from the *edgeR* R-package [40]. Differential genes were identified by fitting negative binomial generalized linear models implemented in *edgeR*. Multiple testing correction was done using the Bonferroni method for the family wise error rate (FWER) [37]. RNA-Seq data were uploaded to the GEO repository under the accession number GSE73857.

### Gene sets and over-representation analysis

Gene sets corresponding to the four WNT networks were created as lists of node labels represented by HUGO gene symbols. Furthermore, additional gene subsets were produced as: 1) the difference of canonical and non-canonical sets, containing only unique canonical genes (can. \ non-can.), 2) the difference of non-canonical and canonical sets, containing genes belonging exclusively to the non-canonical gene set (non-can. \ can.), and 3) the intersection of these two sets (can. ∩non-can.). 4) The last gene set was created as a union of canonical and non-canonical sets (can. ∪non-can.).

Fisher's exact test was performed in order to test for over-representation. When testing for enrichment of a gene set in a list of differential genes a ranking based analysis was performed using Wilcoxon rank-sum test [24].

### Network analysis and visualization

In order to identify differentially regulated WNT modules the up- and down-regulated DEGs were mapped onto the WNT network nodes. In the next step, nodes induced by DEGs were used as terminal nodes for the Steiner tree algorithm as implemented in the SteinerNet R-package [41,42]. In this analysis a minimum spanning tree was identified, which contains all terminal nodes. So-called Steiner nodes are introduced into the tree to ensure its connectivity. Both DEGs and Steiner nodes were used to extract induced modules containing all edges between these nodes. Differentially regulated WNT modules were plotted using the Fruchterman-Reingold layout [43] combined with manual interactive editing utilizing the *tkplot* R-function from the igraph R-package. For visualization purposes the range for color coding of fold-changes was limited to ±3 and ±4 for the modules based on microarray expression data and on RNA-Seq expression data, respectively.

### Modules validation

The differentially regulated modules of WNT networks originating from the public microarray expression data and the newly generated RNA-Seq data were investigated for graph overlaps. Therefore, the *calcGraphOverlap* R-function from the *rBiopaxParser* R-package was utilized and the modules based on microarray data were used as reference graphs.

## Results and Discussion

### Curated and merged WNT sub-pathway networks

Several publicly accessible databases provide WNT pathways to differing extents. We collected 26 pathways linked to WNT signaling and sorted them into four conceptual groups (S1 Table). We defined the first two groups as 1) canonical and 2) non-canonical WNT signaling. However, after stratifying the pathways into these two groups we noticed that several pathways did not fall into this classification. After scrutinizing these pathways more closely, we decided to create two additional groups: for the pathways which act upstream of the WNT cascade we defined 3) the regulation of WNT signaling and for the pathways which reflect the activation state of the β-catenin destruction complex we created 4) the inhibition of canonical WNT signaling as groups.

**Fig 2:**
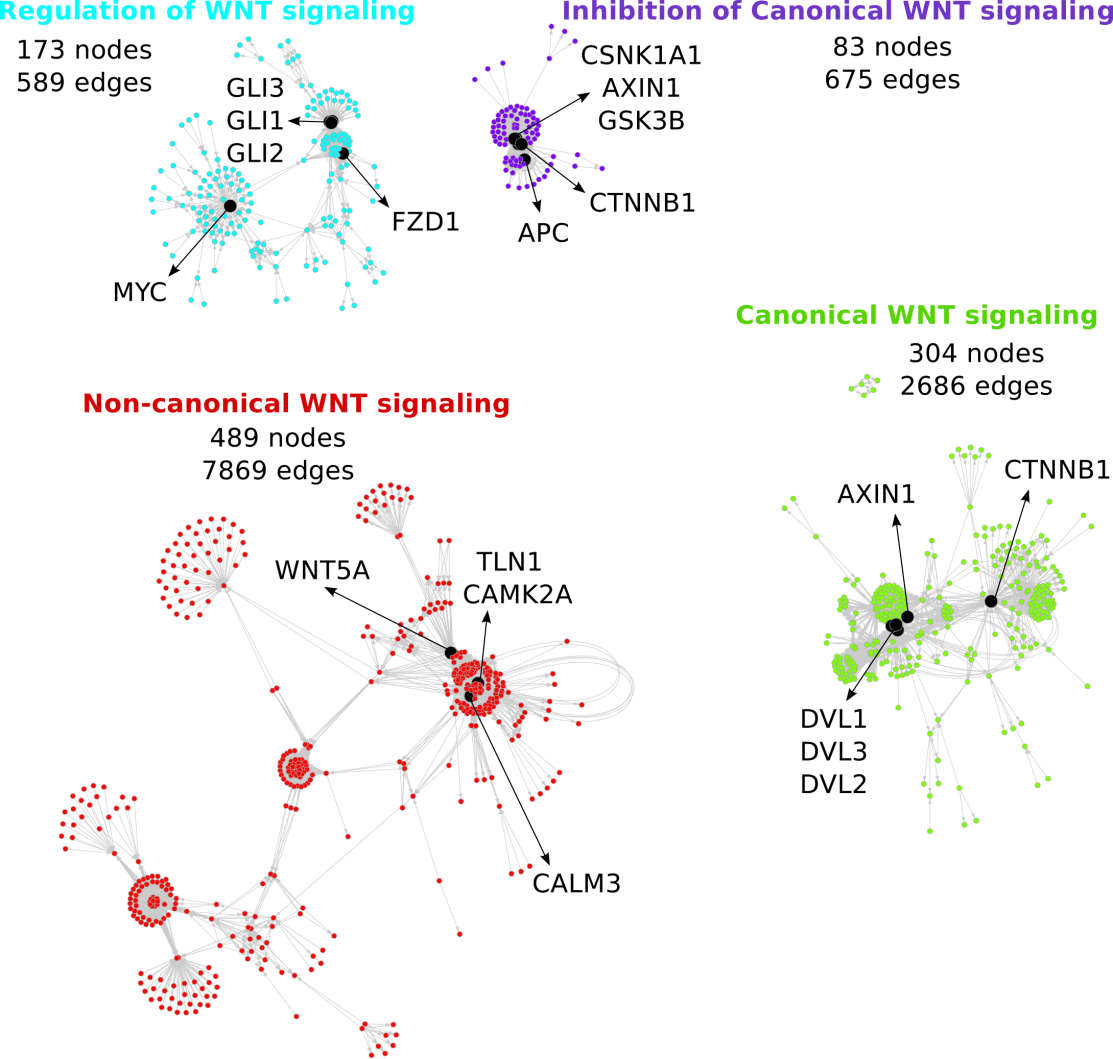
Four WNT networks representing different WNT signaling pathways. The size of each network is defined by the numbers of nodes and edges. The network key nodes are visualized in black. For a more detailed visualization see S1, S2, S3 and S4 Figs.

Within each group, the pathway graphs were merged into a singular signaling network resulting in four distinct networks (S1, S2, S3 and S4 Figs): canonical WNT signaling, non-canonical WNT signaling, inhibition of canonical WNT signaling, and regulation of WNT signaling. For a broader overview of these relatively large, richly interconnected networks, we also visualized them together in a more condensed form (Fig 2). Further, the networks were made available in a simple interaction format-encoded file (S2 Table).

The size of the non-canonical network (489 nodes, 7869 edges) exceeded that of the canonical network (304 nodes, 2686 edges), even though the number of scientific publications seems to be imbalanced in favor of canonical WNT signaling knowledge [44]. The greater size of the non-canonical network is the result of the major data contribution from the Reactome database, more specifically the complex *beta-catenin independent WNT signaling* pathway graph.

In order to create a computationally suitable signaling network model in an automatized and reproducible way, the used pathways were limited to the databases using the BioPAX model. We focus on defining a graphical model of the WNT signaling pathways that can be used in network-integration analysis of static expression data. Our model does not contain any kinetic or mechanistic information. Alternative modeling approaches such as kinetic WNT models [45,46] based on ordinary differential equations were not considered. The databases utilizing the BioPAX format differ in their level of encoded detail. While the BioPAX definition is fixed, the specific syntax used can vary depending on the focus of a particular database provider, that is, ranging from a coarsely encoded 'controlling interaction' to a specific description of binding and phosphorylation mechanisms. This ultimately leads to variations in the granularity of details on the WNT pathway graphs from different databases.

Owing to their considerable size, systematic manual inspection of the networks was challenging. However, before we began integrating the new networks with any experimental data we wanted to assess the quality of these networks. To evaluate their biological soundness, we took two approaches: First, we examined the interconnections of well-established WNT pathway members within our networks. Second, we identified key nodes in the networks which represent central signaling genes.

### Subnetwork of reviewed WNT genes shows quality level of the new networks

**Fig 3:**
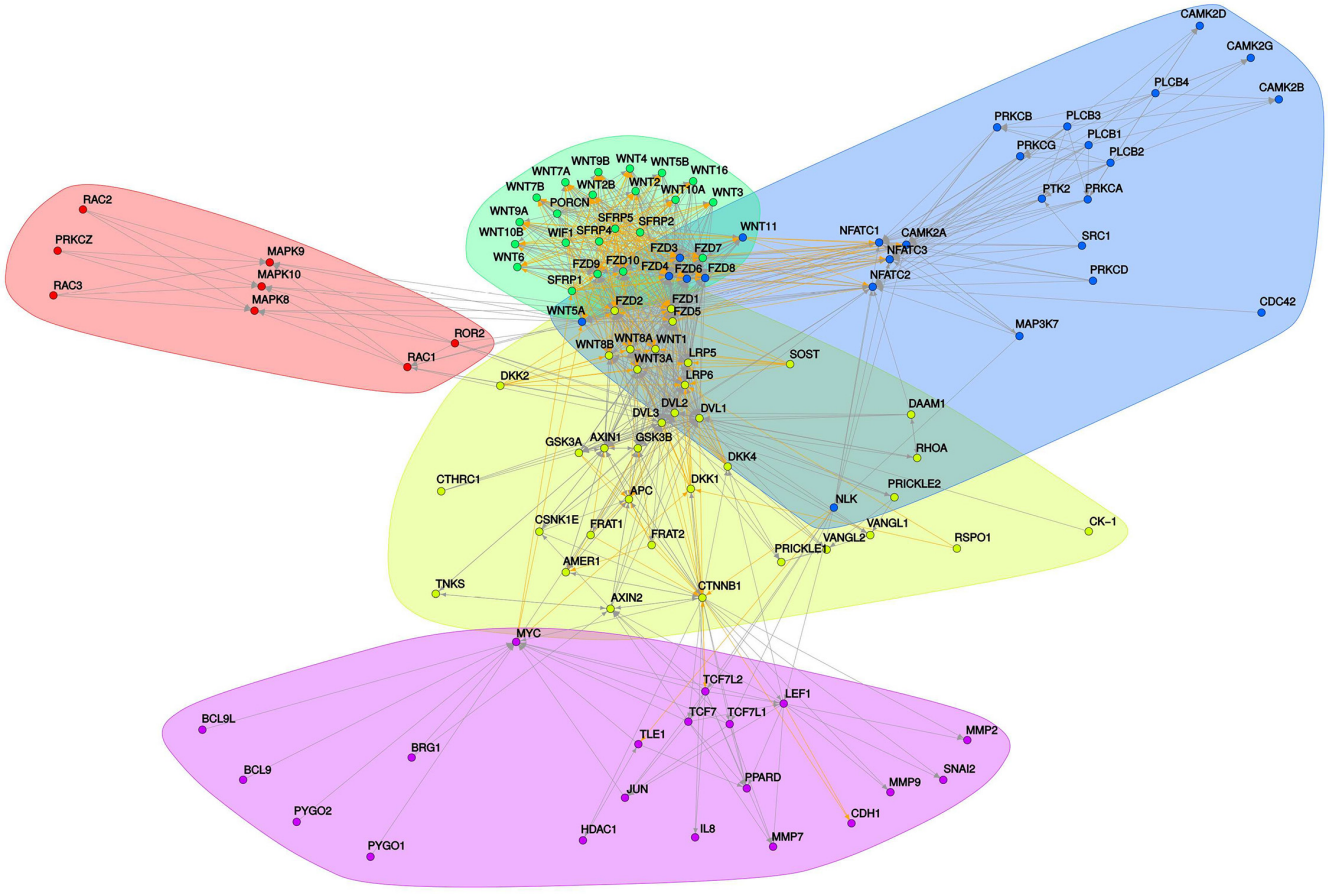
Subnetwork of WNT network genes reviewed in the literature. The five communities are marked in different colors. The directed edges represent activating (gray) and inhibiting (orange) interactions.

In order to ascertain the biological relevance of the newly constructed networks, we were interested in surveying how well-known WNT pathway genes are represented therein. We collected an unbiased list of 121 genes of different WNT sub-pathways reviewed in several publications [3,15,47,48] (S3 Table) and explored how they were interconnected in our networks. The nodes of 112 genes from the list were connected by edges from all four networks resulting in a unified subnetwork (Fig 3). We further clustered the subnetwork, revealing the structure of five densely interconnected communities.

The first community consists mainly of WNT genes and secreted frizzled-related protein ligands. Two communities (Fig 3, blue and red) comprise β-catenin-independent signaling pathways—wherein the smaller community includes players from the PCP pathway (Fig 3, red); the other includes WNT5A and WNT11 and further downstream components of the WNT/Ca^2+^ pathway (Fig 3, blue). Interestingly, Fz receptors are intriguingly distributed among the communities, e.g. FZD3, FZD6 and FZD8, which are associated with β-catenin-independent signaling [44,49], are clustered into the corresponding non-canonical community. The next community is more heterogeneous (Fig 3, yellow), as it contains both canonical (e.g. WNT3A, LRP5/6, AXIN1/2, APC, CTNNB1) and non-canonical (e.g. VANGL1/2, RHOA, DAAM1, PRICKLE1/2) components. Along these lines, the central nodes of this community are formed by DVL isoforms known to transduce both types of WNT signals [50]. Finally, transcriptional effectors and target genes are grouped in another unique community (Fig 3, purple).

However, the limitations of our approach also became apparent: major components of the nuclear transcription complex such as BCL9 and PYGO1/2 are not connected to members of the TCF/LEF family but rather to MYC. In a similar fashion, the ROR2 receptor lacks any connection to a suitable ligand. This stems from the design of how pathway data encoded in the BioPAX model are parsed. While the focus is on controlling mechanisms, the physical binding processes, usually represented by protein-protein interactions (PPI), are not included. Therefore, we do not expect the networks to be the complete representation of cell signaling processes. For a more complex view on the signaling pathways these networks could be further combined with complementary information, such as PPI data.

In conclusion, this subnetwork revealed the relevant topology of the core members of WNT signaling. Moreover, its densely clustered communities and their interconnections reflect established biological models of WNT signaling. Although we reused knowledge from the literature within the subnetwork construction, our approach allowed us to acquire relevant insight into the WNT networks in general.

### Network key nodes correspond to well-recognized WNT players

To further examine the features of the four newly constructed WNT networks we identified their key nodes (Fig 2, nodes depicted in black) to determine central genes. These key nodes were defined as an overlap between the 15 highest scored degree centrality nodes and the 15 highest scored betweenness centrality nodes, assuming important signaling roles of these key players within each of the networks. A maximum number of five top key nodes are visualized for each network.

The top key node in the canonical network is CTNNB1, i.e. β-catenin, the core member of the canonical WNT signaling cascade. The subsequent top key nodes are genes from the DVL protein family DVL1-3 and AXIN1, recognized for their essential role in mediating and modulating β-catenin-dependent WNT signals [3]. The key nodes of the non-canonical network comprise WNT5A, CAMK2A, TLN1 and CAML3. While WNT5A and CAMK2A are known important components of β-catenin-independent signaling [51], the identification of TLN1 as a key node was rather unexpected. This gene encodes Talin-1, which is a major cytoskeletal protein associated with integrin signaling and focal adhesion [52]. Within the “inhibition of canonical WNT signaling” network, the key nodes include genes involved in the β-catenin destruction complex (AXIN1, APC, and CTNNB1) as well as other main negative regulators of canonical signaling, such as GSK3B and CSNK1A1 [47]. In the network for “regulation of WNT signaling”, the top key nodes identified are transcription factors from the hedgehog pathway (GLI1, GLI2, and GLI3) and the MYC gene. These nodes share the ability to modulate WNT signaling by regulating the expression of WNT signaling inhibitors: While Gli1 protein upregulates SFRP1 expression [53], the c-myc protein, which has been shown to be a target of WNT signaling, also activates canonical WNT by suppressing DKK1 and SFRP1 expression [54].

Within this approach we identified key nodes reflecting central pathway players which fall in line with the current understanding of WNT signaling. Given the dependency of our newly created WNT networks on external, prior knowledge, it comes as no surprise that no previously unknown signaling components were identified. However, our approach offers a new perspective on important players and shared mechanisms within distinct WNT sub-pathways that could be amenable to further experimental investigation, one example being Talin-1.

### Gene expression data integration with the WNT networks

We then focused on evaluating whether our newly constructed networks allow for the stratification of WNT sub-pathway activity in gene expression data. To do so, we analyzed a public microarray dataset comprising two breast cancer cell lines: MCF-7 and MDA-MB-231, representing weakly and highly invasive breast carcinomas, respectively. By comparing the expression profiles of these two cell lines and integrating the results of the differential analysis with WNT models, we aimed to further clarify the roles of distinct WNT pathways in the two breast cancer subtypes. Out of the four new WNT networks, we focused on canonical and non-canonical signaling, as our previous results [22] suggest that β-catenin-independent signaling plays a major role in the basal-like subtype of breast cancer. Both the canonical and non-canonical WNT models were utilized for data integration in the form of gene sets as well as signaling networks. While the network models are based on database knowledge, which tends to represent a general cellular context, by adopting this integration approach we aimed to identify specific WNT pathway modules linked to the more invasive phenotype of the MDA-MB-231 cell line.

#### Enrichment analysis

**Fig 4:**
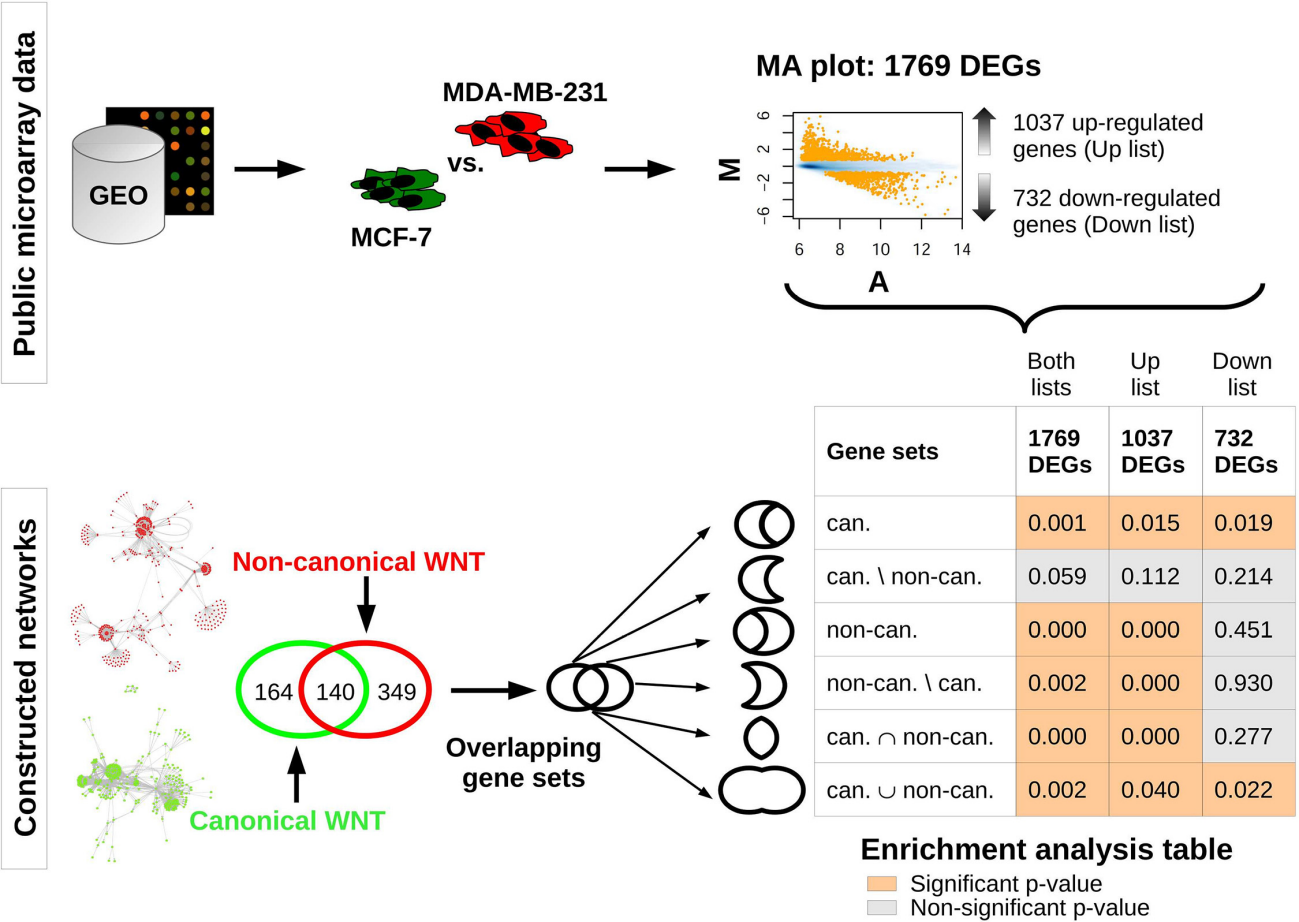
Workflow and delineation of enrichment analysis. Upper part: Public microarray data were retrieved from the GEO repository and expression profiles of MCF-7 and MDA-MB-231 cell lines were compared, resulting in the identification of 1769 significantly differentially expressed genes (DEGs). These genes were separated into two lists: up-regulated and down-regulated genes in the MDA-MB-231 compared to the MCF-7 cell line. Lower part: Canonical and non-canonical network nodes were used to create gene sets represented by HUGO gene symbols. The two gene sets were further split into intersection and unique component subsets and merged into a union gene set. Lists of DEGs (up, down and both lists) were tested for enrichment in the WNT gene sets and subsets. Enrichment was considered significant for p-values < 0.05.

We identified 1769 differentially expressed genes (DEGs) between the cell lines, from which 1037 genes were up-regulated and 732 genes were down-regulated (S4 Table) in MDA-MB-231 compared to MCF-7. The DEGs were tested for over-representation in WNT gene sets originating from the networks (Fig 4). Besides testing the enrichment of canonical and non-canonical gene sets, we were also interested in their subsets. Thus, we created distinct gene sets representing their intersection, union, and relative complements, representing unique genes for each gene set. We found that all 1769 DEGs and up-regulated DEGs were not significantly enriched in the unique canonical subset, while proving to be enriched in the complete canonical gene set as well as in all other tested sets. This suggests that the expression profiles of genes that belong exclusively to canonical WNT signaling do not differ between the two cell lines. Furthermore, the non-canonical gene set and both its subsets (intersection and complement) are enriched in the list of up-regulated DEGs but not in the list of down-regulated DEGs, indicating the enhanced transcription of non-canonical WNT pathway components in MDA-MB-231 compared to MCF-7.

#### Differentially regulated WNT modules

Our next step was to integrate the differential genes between the MDA-MB-231 and MCF-7 cell lines with the graph representations of canonical and non-canonical signaling pathways.

Differentially regulated modules, containing nodes of DEGs and Steiner nodes for graph connectivity, were extracted from canonical and non-canonical networks. In these modules, the DEGs are placed into a context of their signaling interaction partners representing aggressive breast-cancer-specific WNT pathway networks.

**Fig 5:**
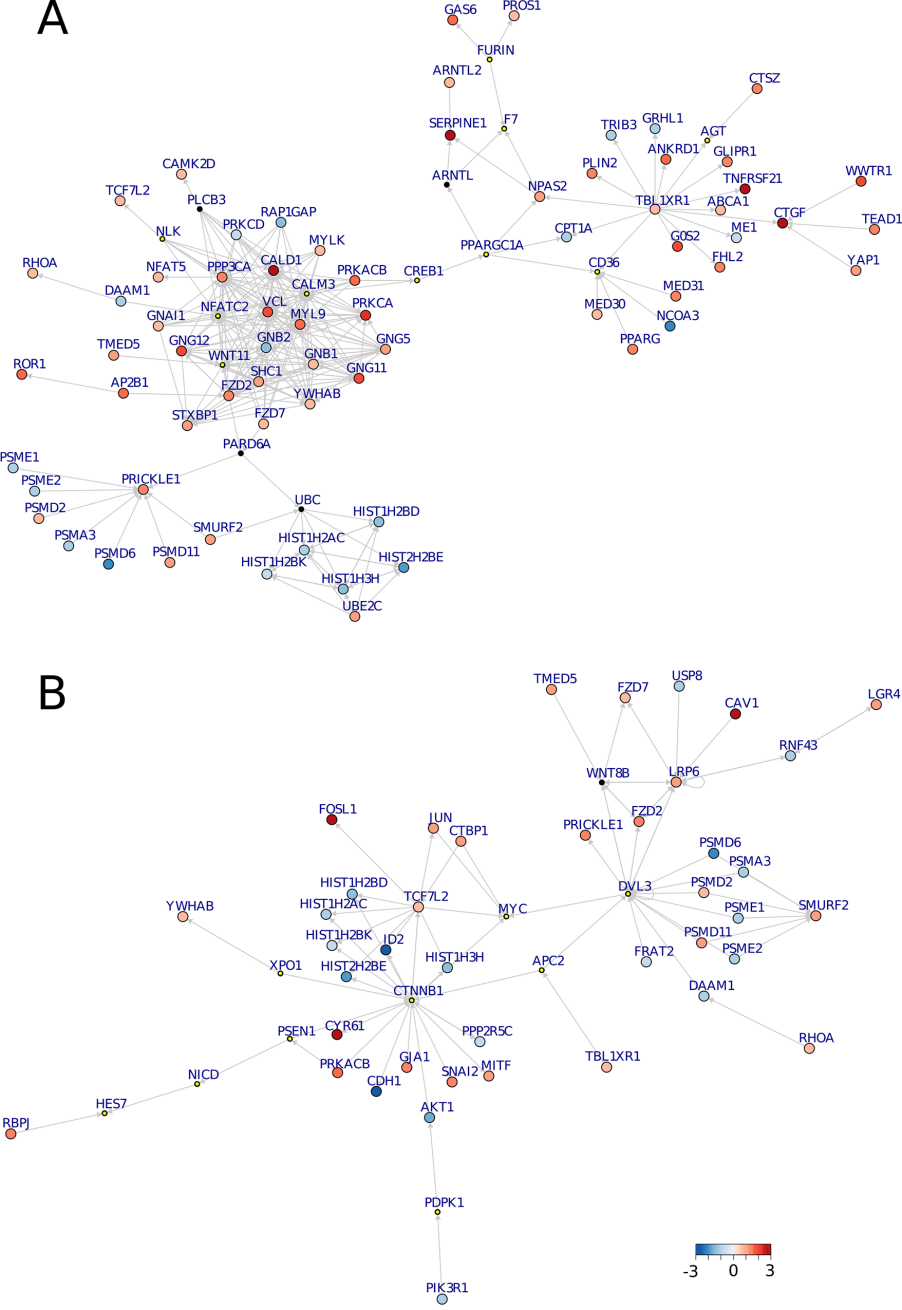
Non-canonical (A) and canonical (B) modules. The color coding of the bigger nodes represent log fold-changes of significantly DEGs: blue represents down-regulation and red up-regulation of the genes in MDA-MB-231 compared to MCF-7. The smaller nodes where introduced by the Steiner tree analysis to ensure connectivity of the differentially regulated WNT modules; the smaller nodes with the yellow dots depict Steiner nodes which were confirmed by the modules in the validation dataset. The directed edges represent controlling interactions.

As expected from the sizes of the original WNT networks and the results of the enrichment analysis, the non-canonical module was greater in size and more richly interconnected than that of the canonical module. The non-canonical module comprised 267 edges and 82 nodes, 68 of which were induced by DEGs and 14 were connecting Steiner nodes (Fig 5A). The canonical module contained 82 edges and 51 nodes, 10 of which were Steiner nodes and 41 induced by DEGs (Fig 5B).

Both the canonical and non-canonical WNT modules demonstrate the importance of Steiner nodes to maintain connectivity of the network. Either these genes are not differentially expressed under the different conditions or are not measured within the microarray experiment at all. Key cancer drivers are often not detected through the analysis of differential expression, but they play central role in networks by interconnecting many genes, which are expression-responsive [55]. In the case of the canonical WNT signaling module, several essential mediators, such as DVL3 and CTNNB1, fall into the first category of Steiner nodes, which genes are not expressed differentially and would not have been revealed by standard differential and enrichment analysis approaches. This also holds true for the down-regulated PI3K/AKT signaling branch that relies on the introduction of PDPK1 as a Steiner node. Within the non-canonical WNT module, an out-branching via members of WNT/Ca^2+^ signaling [56] towards a cluster of edges connected to the transcriptional co-repressor TBL1XR1 can be observed. Depending on its SUMOylation status, TBL1XR1 has been demonstrated as regulating the transcription of β-catenin target genes [57]. While this post-translational modification is not mirrored by our pathway, it certainly constitutes an avenue for further studies.

Thus, the identified WNT modules highlight intriguing events as well as several genes, for which it might prove interesting to test for their biological relevance within the aggressive basal-like breast cancer subtype.

Although we are aware of the absence of some connections in the WNT networks, it is not possible to infer any new edges when adopting this integration approach. However, there are other bioinformatic methods, e.g. nested effect models [58] or Boolean modeling [59], which are able to further infer new network edges based on biological evidence from targeted experiments. Moreover, it may be interesting to combine the WNT networks with complementary knowledge, such as protein-protein interactions (PPIs) or transcription factor-target genes relations. Such approaches may refine the topology of the WNT networks in future.

#### Validation of WNT modules

In order to confirm the findings of our integrative analysis we generated an independent dataset by performing transcriptome sequencing of MCF-7 and MDA-MB-231 breast cancer cell lines. Differential analysis of the RNA-Seq data yielded 6638 DEGs between the cell lines (S5 Table), from which 1344 genes overlapped with the DEGs identified in microarray data. As the RNA-Seq represents a more sensitive technology [60,61], the higher number of DEGs is expected. On the level of fold-changes the 1344 overlap differential genes displayed a 0.78 Pearson correlation (p = 2.2e-16; 95% CI [0.76–0.80]).

We further performed network integration using the RNA-Seq data in the same manner as previously in the microarray application–including enrichment analysis and the identification of differentially regulated modules.

For the purpose of enrichment analysis the Fisher's exact test was not the most suitable in a setting with such a high number of differential genes. Therefore, we preferred the ranking-based enrichment approach utilizing the Wilcoxon rank-sum test. The non-canonical WNT gene set was significantly enriched (p = 0.0198) while the canonical WNT set did not show enrichment (p = 0.3302) in MDA-MB-231 cells. This falls in line with our formulated hypothesis about non-canonical WNT signaling having a predominant role over the canonical WNT pathway in the more aggressive basal-like breast cancer cells In contrast to the microarray expression data, no split of the gene sets into unique subsets was required to recognize this distinction.

Next, the differentially regulated modules of non-canonical and canonical networks were revealed by integration of the WNT networks with the RNA-Seq expression data (S1 File, S6 Table). We refer to these modules as *validation modules*, while their counterparts–non-canonical and canonical WNT modules identified based on microarray data–are referred to as *original modules*.

The non-canonical validation module (Figure A in S1 File) comprised 1332 edges and 192 nodes, from which 13 were introduced as the Steiner nodes. The validation module of the canonical network (Figure B in S1 File) had 367 edges and 108 nodes, from which 10 were Steiner nodes.

We investigated the concordance between the original and validation modules in three ways: 1) by evaluating the graph overlaps on node and edge levels while using the original modules as reference, 2) by assessing the correlation of folds-changes between the overlap genes of the corresponding modules and 3) by inspecting the consistency of Steiner node statuses.

Non-canonical modules showed a node overlap of 75.6% and an edge overlap of 59.5% and a correlation of 0.81 (p = 9.8e-15; 95% CI [0.70–0.89]) for the non-canonical gene fold-changes. Canonical modules exhibited overlaps of 78.4% at the node-level and 70.8% at the edge-level. Their correlation was 0.81 (p = 2.8e-10; 95% CI [0.67–0.90]).

Prominent agreement was further found when focusing on the connecting Steiner nodes of the original modules. In case of the 14 non-canonical Steiner nodes, 10 of them were found in the corresponding validation module: 8 as Steiner nodes (WNT11, NFATC2, NLK, F7, AGT, and CD36) and 2 were detected as significantly differentially expressed genes (FURIN and PPARGC1A). From the 10 Steiner nodes of the original canonical module, 5 were confirmed as Steiner nodes in the validation module (XPO1, CTNNB1, PDPK1, NICD, APC2) and 4 were identified as DEGs (MYC, HES7, DVL3, and PSEN1).

Within the validated Steiner node genes, several have already been investigated in the context of aggressive breast cancer cells or tumors. For instance, NFATC2 was reported to promote breast cancer cell migration and to induce invasion [62]. Similarly, the increased expression of PPARGC1A, transcriptional regulator of multiple metabolic pathways, was associated with the ability of breast cancer cells to metastasize [63,64]. Furthermore, WNT11 was shown as essential for MDA-MB-231 cells motility [65] and its expression was observed in triple-negative breast cancer patients with decreased survival [66].

In summary, the validation modules showed high level of topological as well as transcription concordance with the original differentially regulated WNT modules from the microarray analysis. Moreover, the validated nodes underline the importance of the Steiner nodes within the modules' topology and point to genes with a substantial role in invasiveness of breast cancer cells. The relevance of these nodes would have been neglected by a standard differential analysis without using the signaling network information.

## Conclusions

We constructed computationally suitable network models of WNT signaling cascades in a form of directed signaling graphs and demonstrated in the application example that these WNT networks reasonably reflect distinct WNT sub-pathways. We subsequently integrated the networks with microarray data to better understand the underlying biology of gene expression differences between different breast cancer cell lines. We identified differentially regulated modules representing canonical and non-canonical breast-cancer-specific WNT pathway components, validated these modules in an independent transcriptomic dataset, and highlighted relevant function of topologically-essential elements of the modules.

Following our approach, the intricate WNT networks were shaped into a comprehensive graphical overview of the genes implicated in the aggressive basal-like breast cancer subtype. Utilizing these models as gene sets, we revealed that non-canonical WNT signaling is of importance in this system. Further enhancement of the network topology may be achieved by integration with complementary data types, such as PPIs, to gain even greater insight into pathway signaling processes.

## Supporting Information

S1 Fig Canonical WNT signaling network. (PDF)

S2 Fig Non-canonical WNT signaling network. (PDF)

S3 Fig Inhibition of canonical WNT signaling networks. (PDF)

S4 Fig Regulation of WNT signaling network. (PDF)

S1 File Non-canonical (Figure A) and canonical (Figure B) validation modules. (EPS)

S1 Table Pathways used for the WNT networks construction. The table contains pathway names, source databases and assigned groups for the network construction.
(XLS)

S2 Table Simple interaction format of the four WNT networks. The directed edges are weighted as 1 for activation and as -1 for inhibition interaction.
(XLS)

S3 Table List of 121 genes collected from review publications on WNT signaling. (XLS)

S4 Table Lists of differentially expressed genes from the microarray data. Up- and down- regulated significantly differentially expressed genes in the MDA-MB-231 cell line compared to the MCF-7 cell line.
(XLS)

S5 Table Lists of differentially expressed genes from the RNA-Seq data. Significantly differentially expressed genes in the MDA-MB-231 cell line compared to the MCF-7 cell line.
(XLS)

S6 Table Lists of edges of the canonical and the non-canonical WNT modules and their validation counterpart. (XLS)
