# Supplementary figures and images for: Newly Constructed Network Models of Different WNT Signaling Cascades Applied to Breast Cancer Expression Data
Source: PLoS One. 2015 Dec 3;10(12):e0144014. doi: 10.1371/journal.pone.0144014 (PMC4669165; doi:10.1371/journal.pone.0144014)

# Canonical WNT signaling

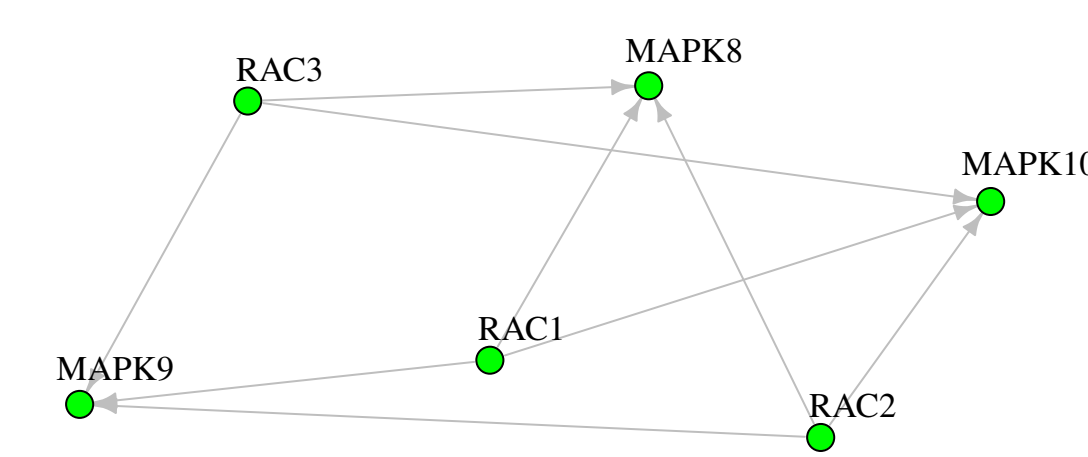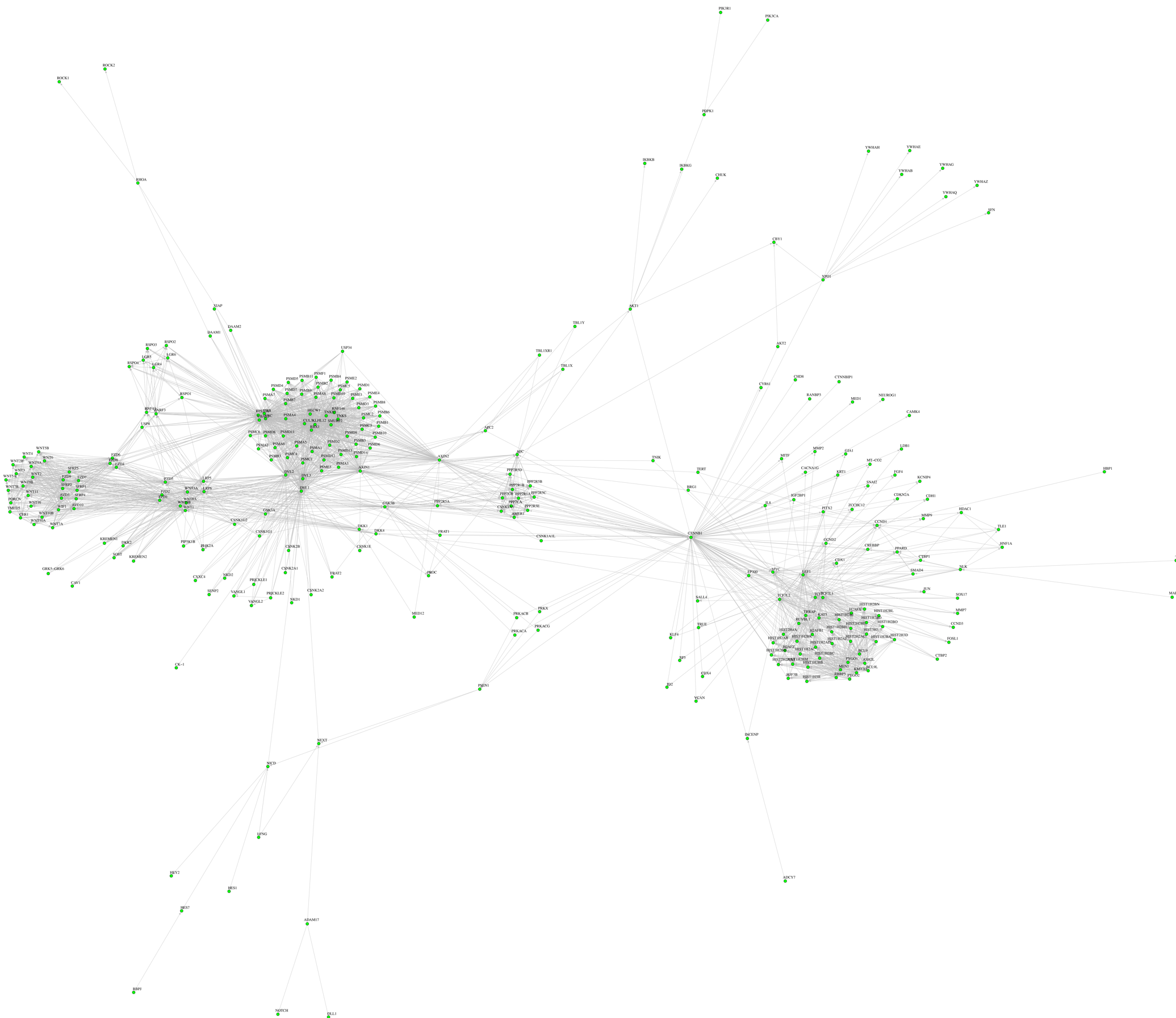

Supplement: S1 Fig — (PDF) [file pone.0144014.s001.pdf]

# Inhibition of Canonical WNT signaling

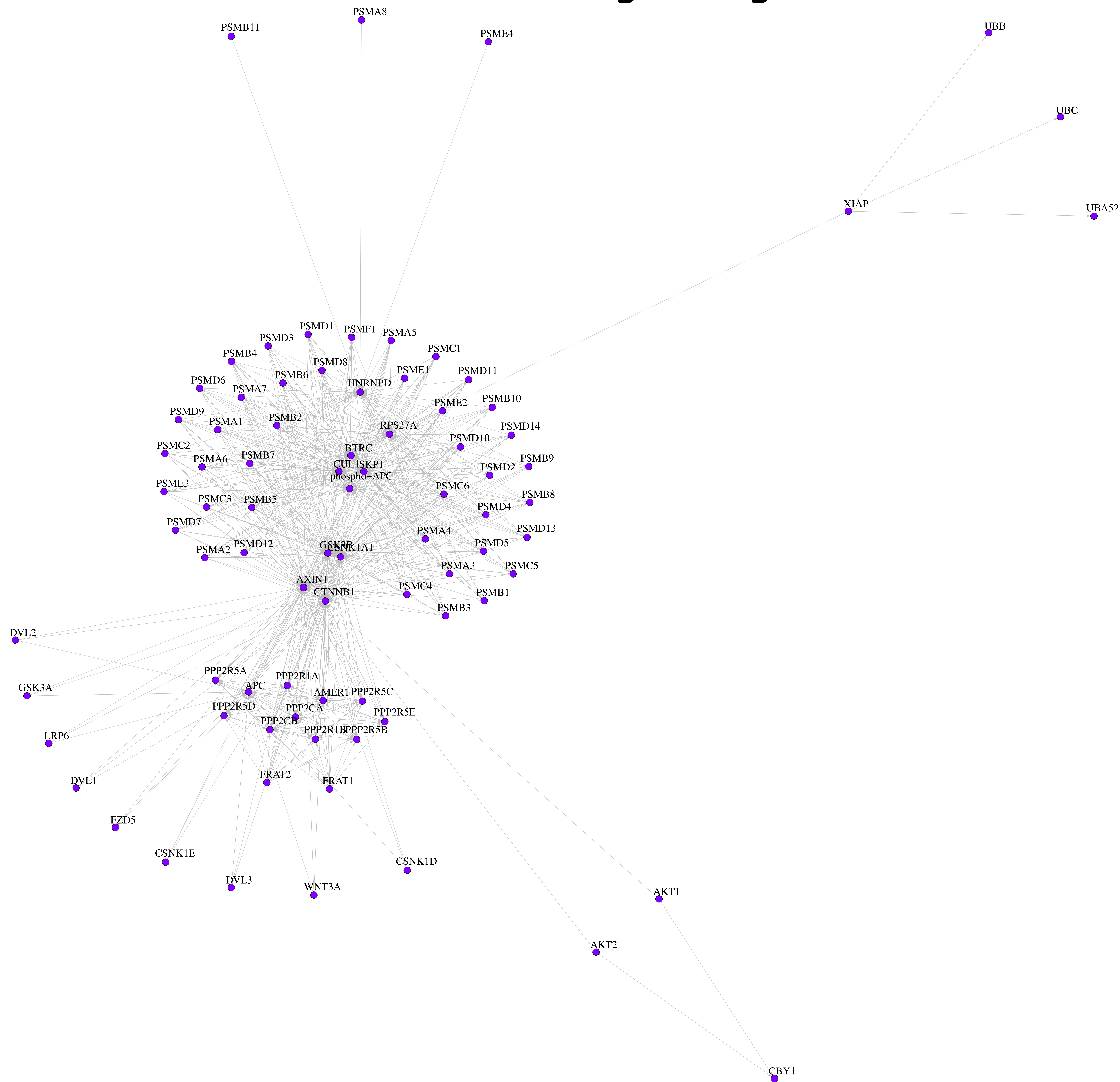

Supplement: S3 Fig — (PDF) [file pone.0144014.s003.pdf]

# Regulation of WNT signaling

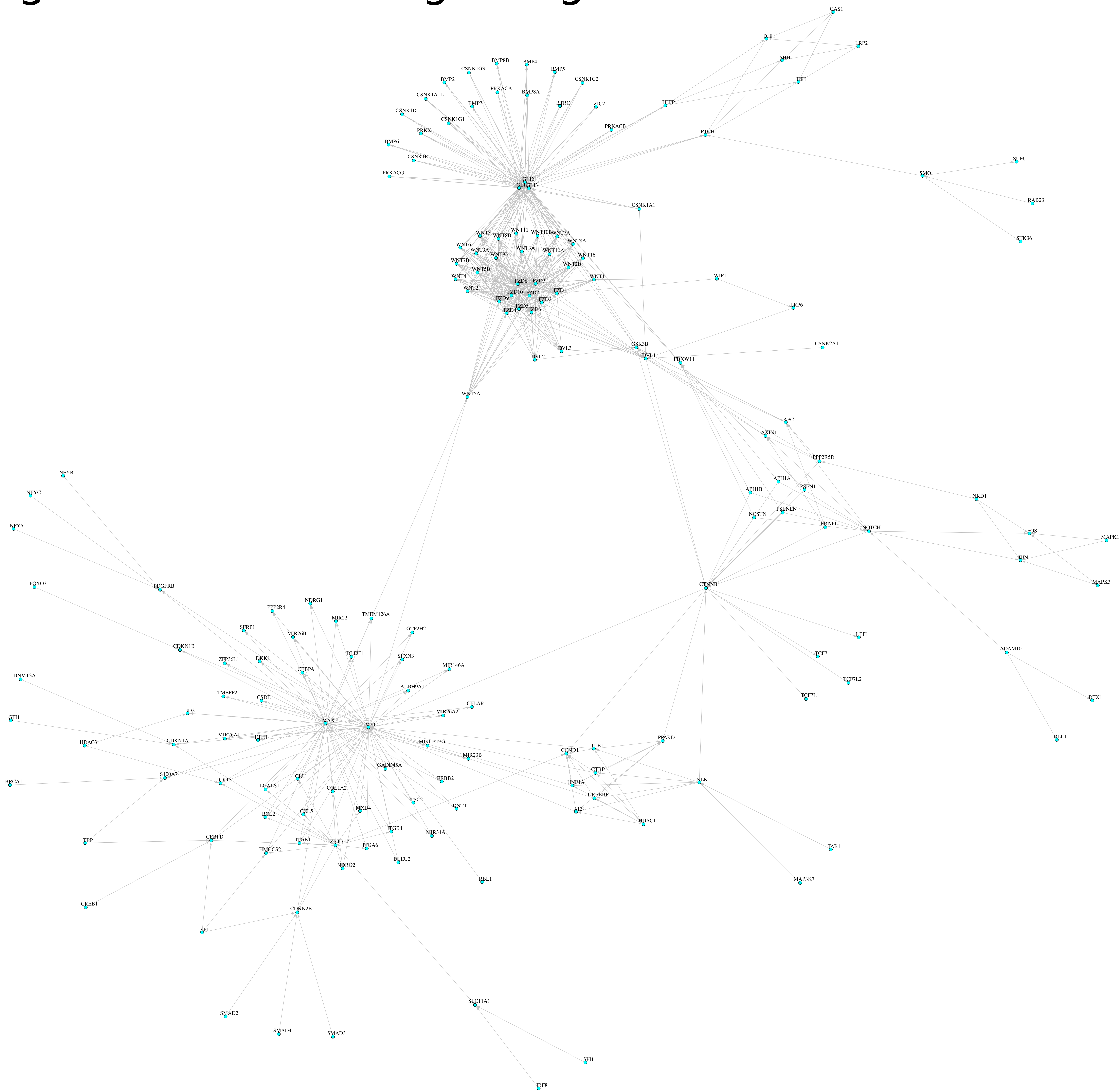

Supplement: S4 Fig — (PDF) [file pone.0144014.s004.pdf]
